# PP2A activation alone and in combination with cisplatin decreases cell growth and tumor formation in human HuH6 hepatoblastoma cells

**DOI:** 10.1371/journal.pone.0214469

**Published:** 2019-04-10

**Authors:** Laura L. Stafman, Adele P. Williams, Raoud Marayati, Jamie M. Aye, Jerry E. Stewart, Elizabeth Mroczek-Musulman, Elizabeth A. Beierle

**Affiliations:** 1 Division of Pediatric Surgery, Department of Surgery, University of Alabama, Birmingham, Birmingham, AL, United States of America; 2 Division of Pediatric Hematology Oncology, Department of Pediatrics, University of Alabama, Birmingham, Birmingham, AL, United States of America; 3 Department of Pathology, University of Alabama, Birmingham, Birmingham, AL, United States of America; Columbia University, UNITED STATES

## Abstract

Despite an increase in incidence, treatments for hepatoblastoma remain virtually unchanged for the past 20 years, emphasizing the need for novel therapeutics. FTY720 (fingolimod) is an immunomodulator approved for use in multiple sclerosis in children that has been demonstrated to have anti-cancer properties in multiple cancer types. We have demonstrated that FTY720 activates PP2A in hepatoblastoma, but does not do so via inhibition of the endogenous inhibitors, CIP2A and I2PP2A, as previously observed in other cancers. PP2A activation in hepatoblastoma decreased cell viability, proliferation, and motility and induced apoptosis. In a subcutaneous xenograft model, FTY720 decreased tumor growth. FTY720 in combination with the standard chemotherapeutic, cisplatin, decreased proliferation in a synergistic manner. Finally, animals bearing subcutaneous hepatoblastoma xenografts treated with FTY720 and cisplatin in combination had significantly decreased tumor growth compared to those treated with either drug alone. These findings show that targeting PP2A with FTY70 shows promise in the treatment of hepatoblastoma and that combining FTY720 with cisplatin may be a novel and effective strategy to better treat this devastating pediatric liver tumor.

## Introduction

Hepatoblastoma is the most common primary liver cancer in childhood, primarily affecting those younger than 5 years of age [1]. The incidence has increased 4-fold in recent years from 1975 to 2007 [2]. Despite an increase in new diagnoses, treatment strategies have not changed significantly in the past 20 years, and outcomes remain poor for those with advanced or recurrent disease. Therefore, novel approaches and therapeutics must be developed to more effectively treat these children.

FTY720 (2-Amino-2-[2-(4-octylphenyl)]-1,3-propanediol, fingolimod) is an FDA-approved immunomodulator used in the treatment of multiple sclerosis [3]. It is a structural analogue of sphingosine and has recently been demonstrated to have anti-cancer properties in a variety of tumor types [4]. In hepatocellular carcinoma, a liver cancer seen primarily in adults, FTY720 has been shown to decrease growth and increase apoptosis both *in vitro* and *in vivo* [5–7]. There appear to be many mechanisms by which these changes are brought about in hepatocellular carcinoma including an increase in caspase-dependent apoptosis and downregulation of phospho-Akt and phospho-Erk-1/2. One of the main mechanisms that may lead to these downstream effects is FTY720-mediated reactivation of protein phosphatase 2A (PP2A).

PP2A is a serine/threonine phosphatase tumor suppressor whose activity is frequently lost in many cancer types [8]. PP2A functions to dephosphorylate proteins, with the most well-defined targets being Akt [9], Erk [10], c-Myc [11] and β-catenin [12], all of which are known to play a role in hepatoblastoma [13–16]. FTY720 has been shown to activate PP2A in preclinical studies of c-Kit-mediated cancers and leukemia [17, 18]. In addition to its effects as a therapeutic agent via activation of PP2A, FTY720 has been shown to act as a sensitizer to traditional chemotherapeutics in colorectal cancer [19, 20].

Due to the anti-cancer properties of FTY720 previously noted in hepatocellular carcinoma and the observation that it sensitized cancers to standard chemotherapeutic agents, we hypothesized that it would also have an anti-cancer effect on the childhood liver cancer, hepatoblastoma, and may be effective in combination with a current standard chemotherapeutic, cisplatin.

## Results

### PP2A was present in human hepatoblastoma cells and FTY720 activated PP2A with no consistent change in the endogenous PP2A inhibitors, CIP2A and I2PP2A

**Fig 1:**
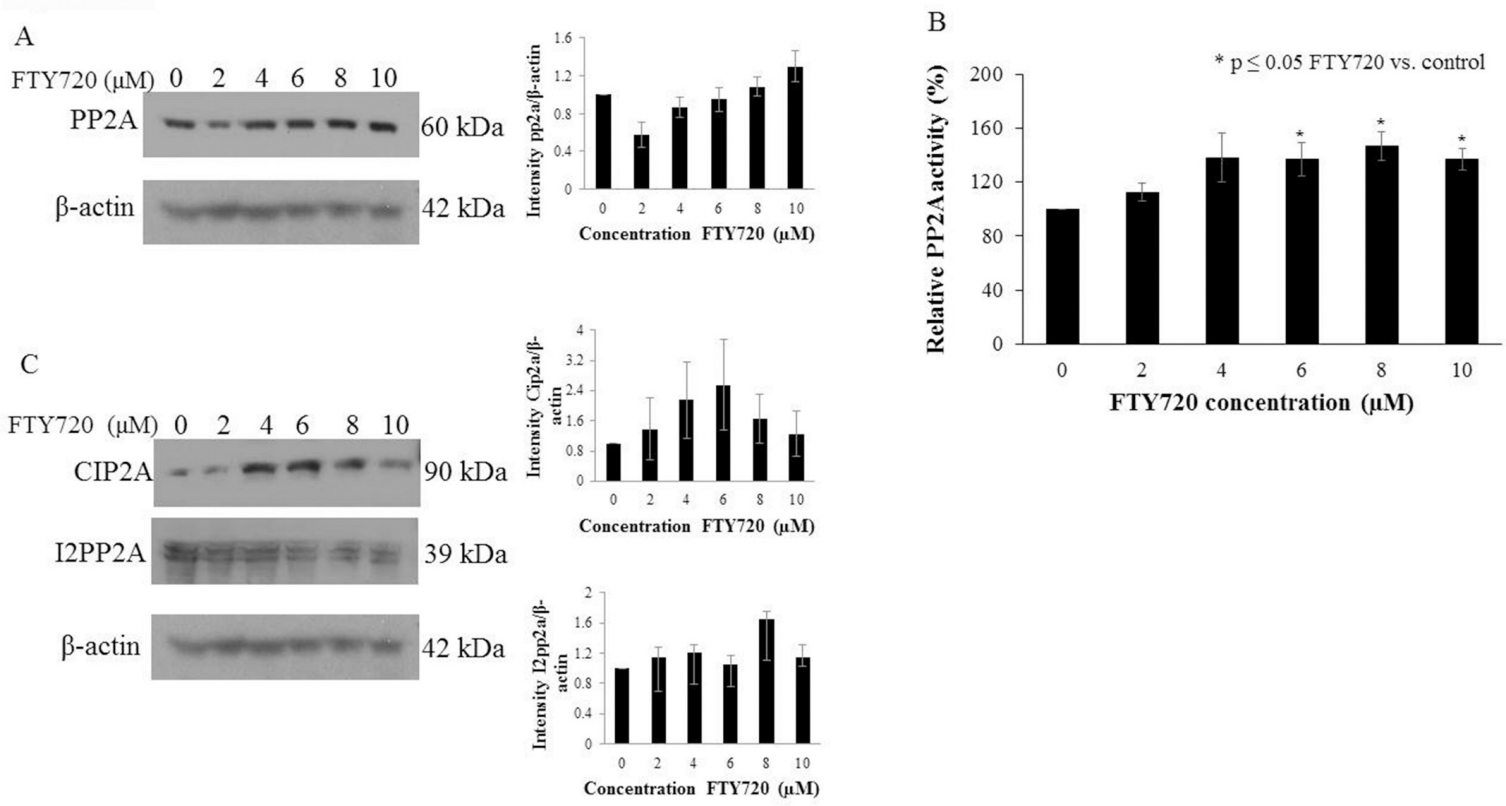
PP2A was expressed and FTY720 activated PP2A in the human hepatoblastoma cell line, HuH6. (A) Immunoblotting for PP2A was performed on HuH6 cell lysates with β-actin serving as a control. PP2A expression was unchanged in the presence of FTY720. Densitometry values relative to actin are listed below each blot. (B) PP2A activity significantly increased following treatment with FTY720 for 24 hours (p ≤ 0.05). (C) Immunoblotting for the endogenous inhibitors of PP2A –CIP2A and I2PP2A –in the presence of increasing doses of FTY720. CIP2A expression increased at lower concentration and returned to baseline at higher concentrations. I2PP2A expression did not change significantly with FTY720 treatment. Histograms are representative of densitometry analysis of three or more biologic replicates and are reported as intensity based upon the respective control that is set at 1. Data are reported as mean ± SEM.

Using immunoblotting, we demonstrated that PP2A was present in human hepatoblastoma HuH6 cells (Fig 1A), and did not significantly change with FTY720 treatment. FTY720 activated PP2A with a 37% increase in PP2A activity in cells treated with 10 μM FTY70 versus untreated cells (Fig 1B). Others have suggested that a mechanism of FTY720-mediated PP2A activation is inhibition of the endogenous inhibitors of PP2A –Cell Proliferation Regulating Inhibitor of Protein Phosphatase 2A (CIP2A) and Inhibitor-2 of Protein Phosphatase-2A (I2PP2A) [19, 21], so we examined the effect of FTY720 on CIP2A and I2PP2A expression. CIP2A expression showed some increase at lower concentrations of FTY720, but returned to baseline expression at higher concentrations; with no significant change. There was no significant change in I2PP2A expression with FTY720 treatment (Fig 1C).

#### FTY720 decreased proliferation, viability, and motility and increased apoptosis in human hepatoblastoma cells

**Fig 2:**
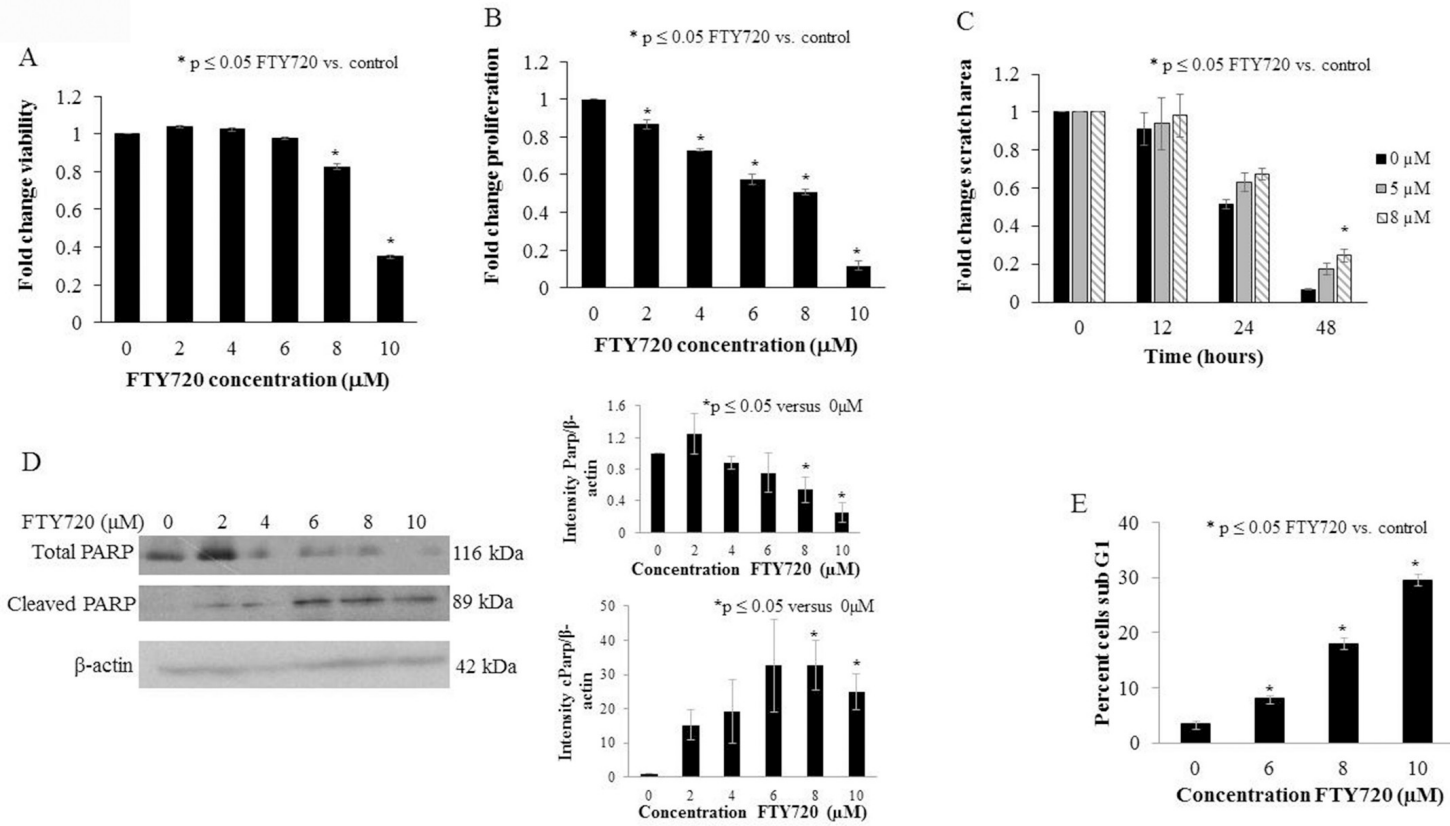
FTY720 decreased viability, proliferation, and motility and induced apoptosis in human hepatoblastoma cells. (A) Following 24 hours of treatment with FTY720, the viability of HuH6 cells measured using the alamarBlue cell viability assay was significantly decreased (LD_50_ = 8.4 μM, p ≤ 0.05). (B) Following 24 hours of treatment with FTY720, the proliferation of HuH6 cells measured using the CellTiter 96 cell proliferation assay was significantly decreased (IC_50_ = 6.4 μM, p ≤ 0.05). (C) HuH6 cells were plated and allowed to reach 80% confluence, at which time the media was changed for fresh media (0, 5, or 8 μM FTY720) and a standard scratch wound was made. At 48 hours, there was a significant decrease in motility in the HuH6 cells treated with 8 μM FTY720 compared to control cells. Data were reported as mean fold change in scratch area remaining ± SEM. (D) Immunoblotting was performed on HuH6 lysates for total and cleaved PARP with β-actin controls. Total PARP decreased and cleaved PARP increased with FTY720 treatment, indicating apoptosis. Histograms are representative of densitometry analysis of three or more biologic replicates and are reported as intensity based upon the respective control that is set at 1. Data are reported as mean ± SEM. (E) Cell cycle analysis was utilized to determine the percentage of cells in the sub G1 phase, representing the apoptotic cell population. There was a significant increase in the percent of hepatoblastoma cells in sub G1 with increasing concentrations of FTY720.

We next examined the effects of FTY720 treatment on hepatoblastoma *in vitro*. Viability was significantly decreased with FTY720 treatment at 8 μM (p ≤ 0.05 vs. control, Fig 2A), and proliferation was significantly inhibited at 2 μM (p ≤ 0.05 vs. control. Fig 2B). The LD_50_ was 8.4 μM and the IC_50_ was 6.4 μM. FTY720 significantly decreased cell motility with fold change scratch area remaining at 48 hours of 24.6% ± 3.3% vs. 6.6% ± 0.3% for FTY720 8 μM vs. control (p ≤ 0.05, Fig 2C). Additionally, FTY720 induced apoptosis in hepatoblastoma cells as evidenced by decrease in total PARP and an increase in cleaved PARP with increasing concentrations of FTY720 (Fig 2D). Examination of the sub G1 population was performed as a second assay to examine apoptosis. There was a significant increase in the cell count in the sub G1 population after FTY720 treatment further indicating apoptosis (Fig 2E, S1 Fig).

#### FTY720 decreased Akt, Erk, and c-Myc signaling

**Fig 3:**
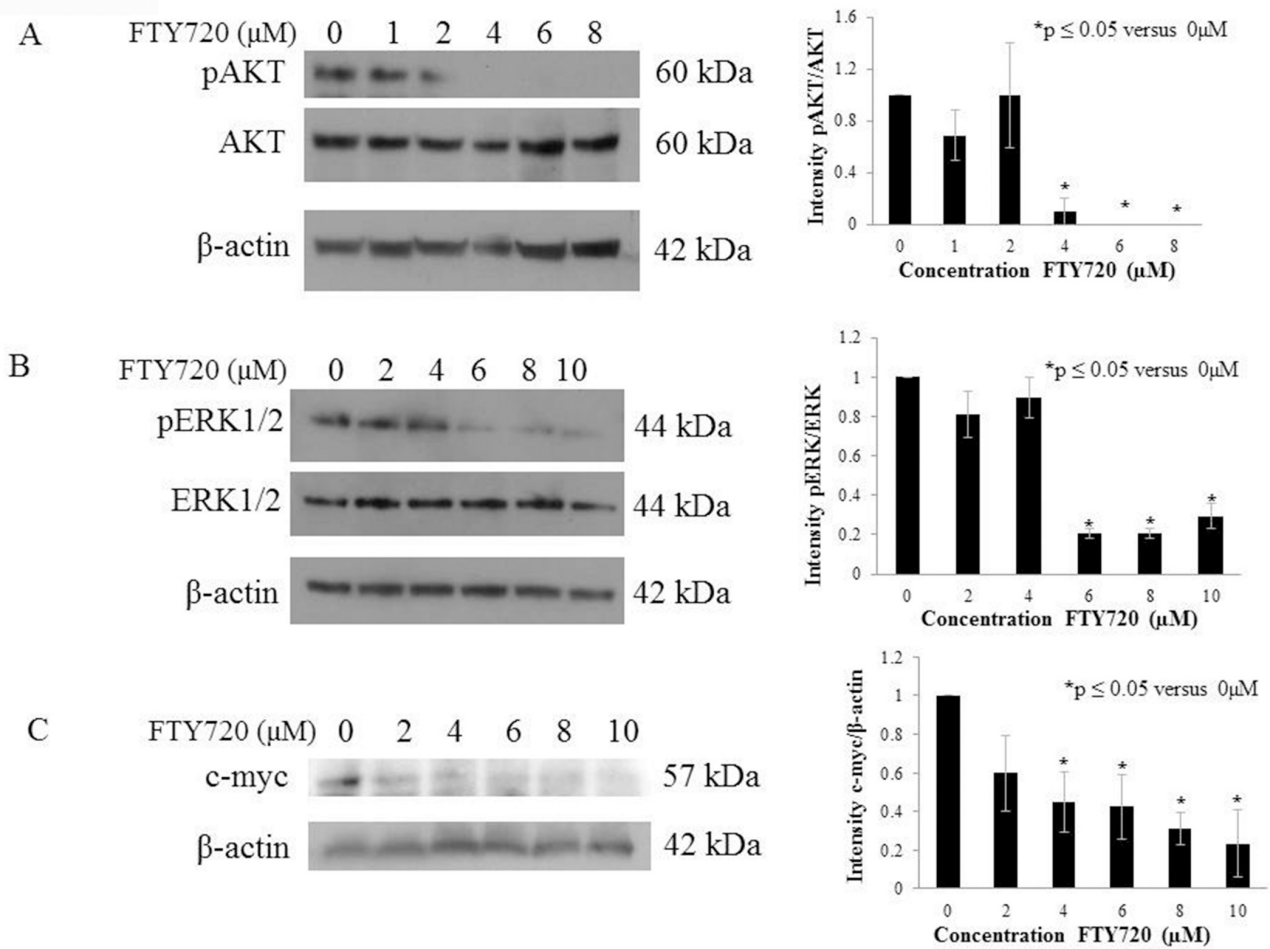
FTY720 treatment decreased phosphorylation of AKT, ERK, and c-myc. (A) Immunoblotting for the activated form of AKT–phospho-AKT (Ser473)–and total AKT revealed a decrease in phospho-AKT with no accompanying change in total AKT, indicating a decrease in AKT signaling with FTY720 treatment. (B) Immunoblotting for the activated form of ERK1/2 –phospho-ERK1/2 (Thr202/Tyr204, Thr185/Tyr187)–and total ERK1/2 revealed a decrease in phospho-ERK1/2 with no accompanying change in total ERK1/2, indicating a decrease in ERK signaling with FTY720 treatment. (C) Immunoblotting for c-myc revealed a decrease in c-myc expression with FTY720 treatment. Histograms are representative of densitometric analysis of three or more biologic replicates and are reported as intensity based upon the respective control that is set at 1. Data are reported as mean ± SEM.

Since FTY720 affected hepatoblastoma proliferation, viability, motility, and apoptosis, we next examined potential signaling pathways that may be involved in these changes. As others have demonstrated that PP2A negatively regulates the Akt, Erk, and c-Myc pathways via dephosphorylation [9–11], we focused our investigation on these targets. Phospho-Akt (Ser473) was decreased with FTY720 treatment whereas total Akt was unaffected (Fig 3A). There was also a decrease in Erk1/2 phosphorylation (Thr202/Tyr204) with no change in total Erk (Fig 3B). Finally, the c-Myc pathway was also affected with a decrease in c-Myc protein expression with FTY720 treatment (Fig 3C).

#### FTY720 decreased hepatoblastoma tumor growth in vivo

**Fig 4:**
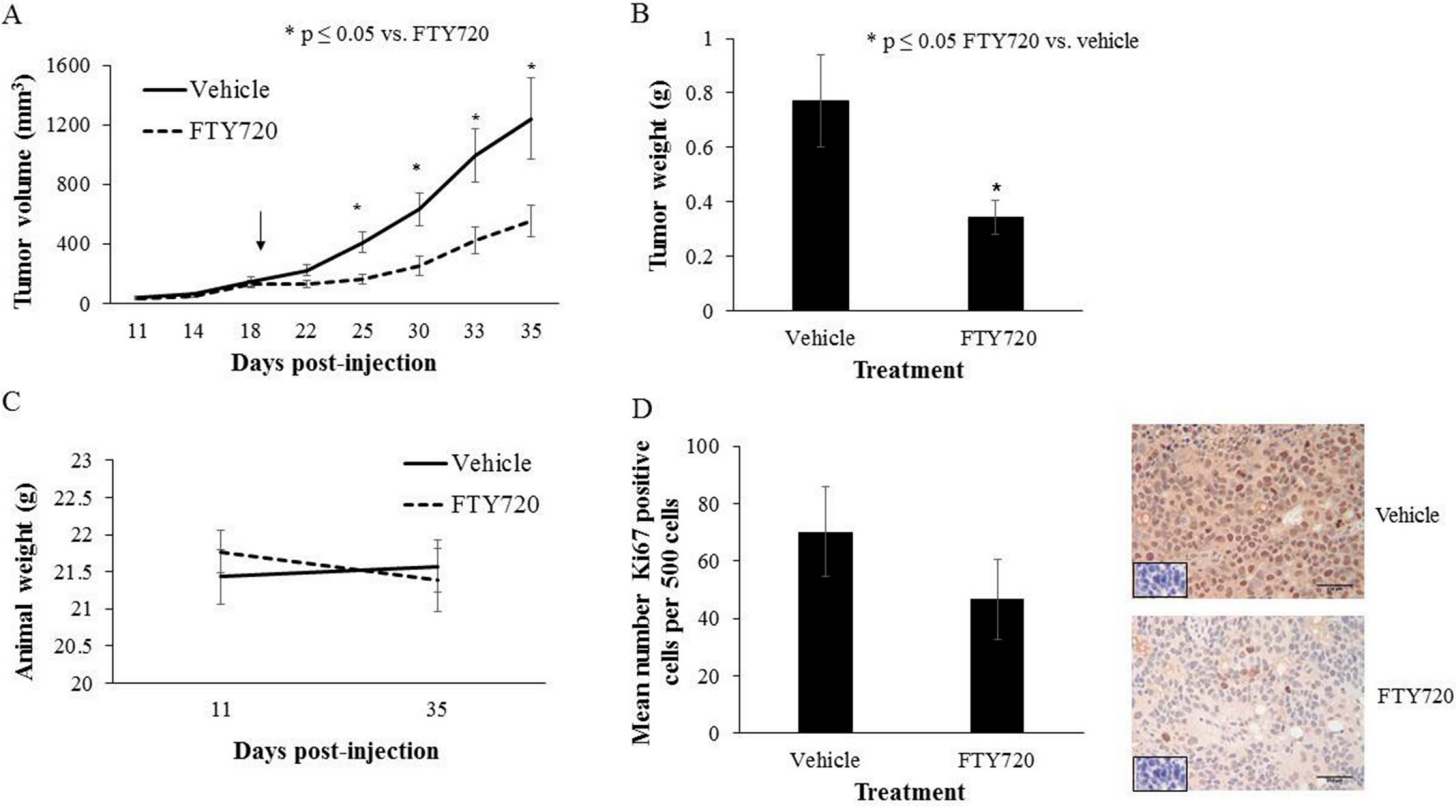
FTY720 treatment decreased tumor growth in a xenograft model of hepatoblastoma. (A) HuH6 cells (2.5 x 10^6^ cells in 25% Matrigel) were injected subcutaneously into the right flank of 6-week-old female athymic nude mice. When tumors reached an average of 150 mm^3^, mice were randomized to receive vehicle (ORA-Plus, 50 μL, n = 7) or FTY720 (10 mg/kg body weight/day in ORA-Plus, 50 μL, n = 7) by oral gavage for 16 days. Animals treated with FTY720 (dashed line) had significantly smaller tumors than those treated with vehicle alone (solid line). The vertical arrow depicts the number of days post-injection on which treatment began. (B) Tumors were weighed at the time of animal euthanasia. Tumors from the FTY720-treated group weighed significantly less than those from the vehicle-treated group. (C) Mice were weighed at the beginning of the experiment and at the time of euthanasia. There was no significant difference in animal weights between those treated with FTY720 and vehicle. (D) Formalin-fixed, paraffin-embedded tumors were used for immunohistochemical analysis of Ki67, a marker of cell proliferation. The number of Ki67 positive cells per 500 cells in a representative section of each tumor was counted. The mean ± SEM for each treatment group were calculated and depicted in a bar graph. Representative photomicrographs of vehicle-treated (*top panel*) and FTY720-treated (*bottom panel*) tumors are shown with IgG control in the bottom left corner of each image. There was a trend toward decreased Ki67 staining in FTY720-treated tumors (p = 0.29). Black bar in lower right corners represents 250 μm.

The effects seen *in vitro*, prompted an investigation of the effects of FTY720 on human hepatoblastoma *in vivo*. Fourteen mice were injected with HuH6 human hepatoblastoma cells subcutaneously into the right flank. They were randomized to receive FTY720 (10 mg/kg body weight/day, N = 7) or ORA-Plus vehicle (N = 7) by oral gavage for a total of 16 days. Mice treated with FTY720 had significantly smaller tumor volumes compared to those given the vehicle only (553.7 ± 105.3 mm^3^ vs. 1242.6 ± 274.7 mm^3^, respectively; p ≤ 0.05; Fig 4A). Additionally, tumors from mice treated with FTY720 weighed significantly less than those from vehicle-treated mice (Fig 4B). The FTY720 treatment was well-tolerated by the animals and the weights of the FTY720-treated mice were not significantly different than the weights of vehicle-treated mice (Fig 4C). While not statistically significant, there was a trend toward decreased proliferation in the tumors from mice treated with FTY720 versus vehicle (46.6 ± 13.8 vs. 70.2 ± 15.8 Ki67 positive cells/500 cells, respectively, p = 0.29; Fig 4D).

#### FTY720 potentiated the activity of the standard chemotherapeutic cisplatin

**Fig 5:**
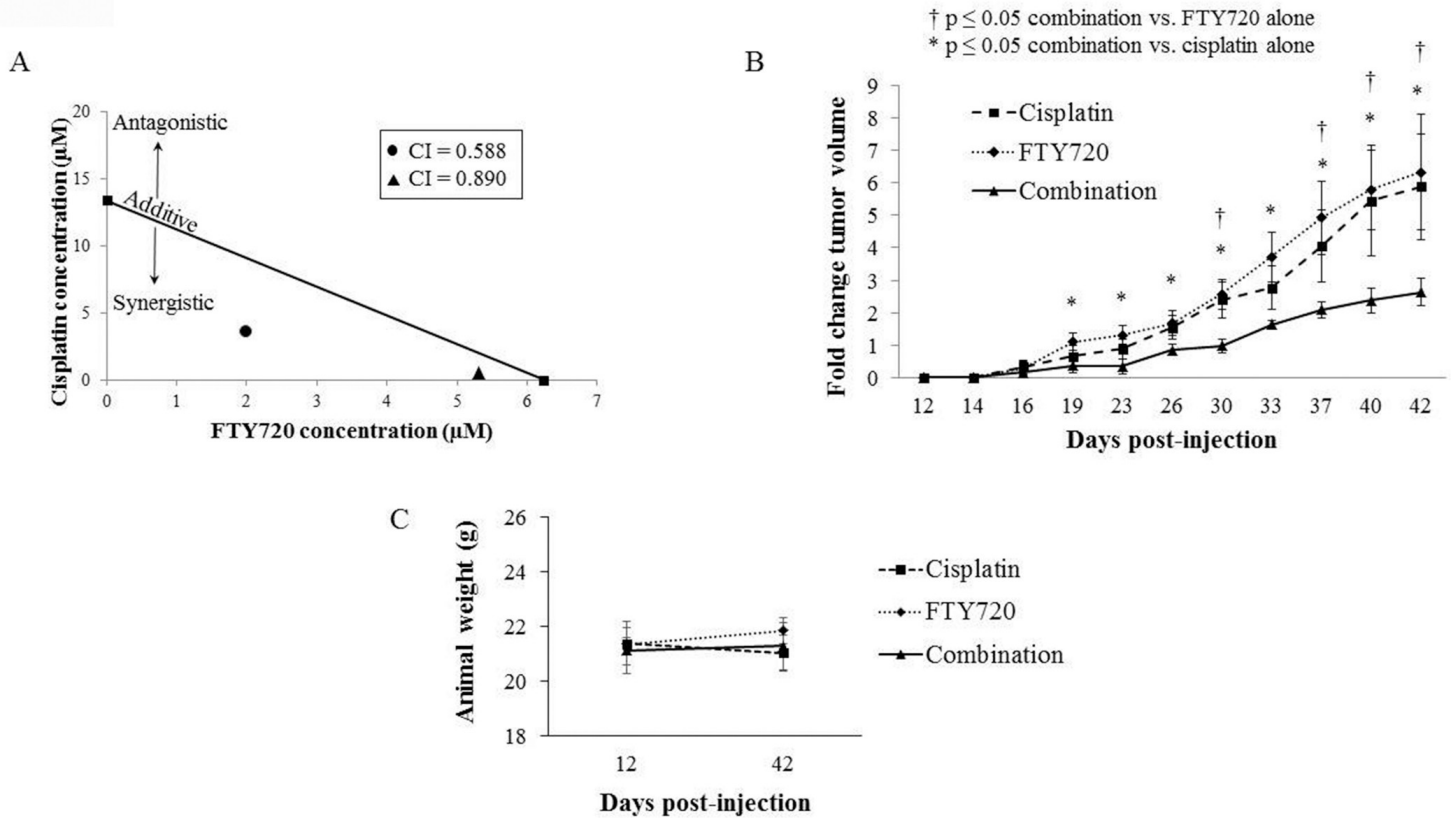
FTY720 potentiated the activity of cisplatin. (A) HuH6 cells were treated with FTY720 and cisplatin combined for 72 hours and cell proliferation measured with the CellTiter 96 assay. Combination indices (CI) were calculated and isobolograms constructed. The straight line connects the IC_50_ of each monotherapy. CI values above the line indicate an antagonistic effect whereas those below the line indicate a synergistic effect between the two drugs. Both CI values were less than 1 (0.588 and 0.890), indicating that the combination of FTY720 and cisplatin was synergistic. (B) HuH6 cells (2.5 x 10^6^ cells in 25% Matrigel) were injected subcutaneously into the right flank of 6-week-old female athymic nude mice. When tumors reached an average of 75 mm^3^, mice were randomized to receive FTY720 (10 mg/kg body weight/day by oral gavage in ORA-Plus, 50 μL, n = 7), cisplatin (2 mg/kg body weight/day by intraperitoneal injection, 100 μL, n = 6), or a combination of the two drugs given at the same doses as monotherapy (n = 6). Animals treated with the combination therapy (solid line) had significantly smaller tumors than those treated with cisplatin (dashed line) or FTY720 (dotted line) alone. (C) Mice were weighed at the beginning of the experiment and at the time of euthanasia. There was no significant difference in animal weights between those treated with cisplatin, FTY720, or combination therapy.

As others have shown that FTY720 acts a sensitizer to standard chemotherapeutics in colorectal cancer [19, 20], we wished to determine whether combining FTY720 with a standard chemotherapeutic for hepatoblastoma would yield a synergistic effect. Cisplatin is currently utilized as single-agent therapy or a component of therapy for all but very low-risk hepatoblastoma cases [22]. The combination of FTY720 and cisplatin treatment was applied for 72 hours to HuH6 cells and proliferation was assessed. Using the Chou-Talalay method [23], an isobologram was constructed and combination indices were calculated (Fig 5A). The combination of FTY720 and cisplatin yielded combination indices less than 1, indicating synergy between the drugs. The indication of synergy between FTY720 and cisplatin *in vitro*, led us to move to an *in vivo* model. Nineteen female athymic nude mice were injected with HuH6 human hepatoblastoma cells subcutaneously into the right flank. Once tumors reached an average of 75 mm^3^, the animals were randomized to receive FTY720 (10 mg/kg body weight/day, N = 7), cisplatin (2 mg/kg body weight/day, N = 6), or combination treatment with FTY720 and cisplatin (N = 6) in the same doses as the single agent for 27 days (S2 Fig). Tumors in the mice receiving monotherapy were larger than those in mice receiving combination therapy (Fig 5B). The animals’ weights at the start and end of the treatment period were not significantly different between groups (Fig 5C).

## Discussion

Despite an increase in hepatoblastoma incidence, treatment has not changed significantly in the past 20 years. Therefore, the identification of new targeted therapeutics, particularly for use in combination with standard therapeutics, is necessary to better treat hepatoblastoma. Repurposing previously approved compounds is an ideal strategy as the regulatory process is less complex, potentially making the time to reach clinical use shorter. We have demonstrated that FTY720, a drug currently approved for use in pediatric multiple sclerosis, has an anti-tumor effect in hepatoblastoma and potentiates the effect of the standard chemotherapeutic cisplatin.

FTY720 was originally developed as an immunosuppressive drug as it decreases the number of peripheral circulating lymphocytes [24]. However, it has since been investigated in a number of conditions and is FDA approved for use in relapsing forms of multiple sclerosis [25]. It has also been examined in preclinical studies as an anti-cancer therapy for multiple cancer types including hepatocellular carcinoma [6]. This study is the first to examine the effect of FTY720 on human hepatoblastoma.

The mechanisms by which FTY720 exerts its effects are diverse and have not been fully defined. The most well-defined mechanism by which FTY720 modulates the immune system is via sphingosine-1-phosphate signaling in which FTY720 binds to sphingosine-1-phosphate receptor, causing internalization of the receptor, which is essential for movement of T cells into the periphery [26]. Therefore, FTY720 causes T cells to remain in the lymphoid organs where they cannot exert their immune effects on peripheral tissues. We focused on the non-immune-mediated mechanisms, which are the main pathways affected at doses greater than 2 μM and those which are involved in the anti-cancer properties of FTY720 [27]. By utilizing long-term passaged human hepatoblastoma cells *in vitro* and athymic nude mice, which lack T cells, for the *in vivo* experiments, we were able to focus on these non-classical, non-immune pathways.

PP2A is a tumor suppressor that is activated by FTY720, either directly or by inhibition of the inhibitors of PP2A –CIP2A and I2PP2A [19, 21]. While we did observe PP2A activation with FTY720 treatment in hepatoblastoma, we found no significant change in I2PP2A expression. In addition, while CIP2A expression increased at lower concentrations of FTY720, it returned to baseline expression at higher concentrations. These findings indicated that decreased expression of the inhibitors of PP2A was likely not the mechanism by which FTY720 activated PP2A in HuH6 hepatoblastoma cells. It remains possible that FTY720 interferes with the interaction between PP2A and its endogenous inhibitors as others have found in leukemia [28], yielding PP2A activation, or FTY720 may directly activate PP2A. Further delineation of these potential mechanisms will be the subject of future investigations.

Given that PP2A is a tumor suppressor, it follows that activation of PP2A would lead to anti-tumor effects. Researchers have found that PP2A inactivation promotes the development of multiple cancer types including breast [29], colorectal [19], and oral cancer [30] resulting in studies to determine whether PP2A activation may serve as a potential therapeutic in breast [29], oral [30], and prostate cancers [31]. However, no studies have previously examined the role of PP2A in hepatoblastoma. We found that FTY720 decreased viability, proliferation, and motility and increased apoptosis in hepatoblastoma cells *in vitro*. We explored potential downstream signaling pathways known to be involved in hepatoblastoma [14–16] and noted that Akt and Erk were dephosphorylated and c-myc expression was decreased with FTY720 treatment; findings similar to what other investigators have demonstrated with PP2A activation [9–11]. These findings indicating the promiscuity of PP2A could be perceived by some to be unfavorable. However, the ability to target cancer cells through multiple pathways with a single agent may prove to be advantageous as it decreases multiple pro-tumor pathways in parallel.

The lack of multiple available cell lines in which to conduct these studies was one limitation to the ability to draw conclusions to the relevance of these data in the human condition. However, multiple authors have demonstrated certain molecular dysregulations associated with poor prognosis hepatoblastoma. One such mutation is the high expression of Polo-Like Kinase 1(*PLK1*) oncogene. Yamada and colleagues showed that PLK1 mRNA was significantly more abundant in hepatoblastomas compared to normal liver specimens and was associated with a significantly poorer 5 –year survival [32]. Investigators have shown that FTY720 targets Plk1 signaling in prostate cancer cells and may also affect the Wnt/beta-catenin pathway through its action on Plk1 [33]. Mutations in *CTNNB1* which encodes for beta-catenin protein have also been recognized as a marker of poor prognosis in hepatoblastoma [34]. Therefore, there is evidence that the PP2A environment may be dysregulated in poor prognosis hepatoblastoma, and FTY720 may have a significant impact on poor prognosis hepatoblastoma tumors through targeting both the Plk1 axis and the Wnt/beta-catenin pathway via effects on PP2A. With the ultimate goal being the translation of these findings to the clinical arena, we progressed to an *in vivo* model. FTY720 treatment yielded significantly decreased tumor growth compared to vehicle in mice bearing HuH6 tumors. We chose to implant the cells in a heterotopic location to allow more frequent monitoring of tumor growth. No transgenic mouse model exists to study hepatoblastoma specifically as all of the transgenic animals develop mixed hepatoblastoma and hepatocellular carcinoma [35]. Only one chemically-induced hepatoblastoma model exists, using diethylnitrosamine and sodium phenobarbital, but again, the tumors phenotypically express elements of both hepatoblastoma and hepatocellular carcinoma. The xenograft model is thus one of the best methods currently available to study hepatoblastoma *in vivo* as it allows for the study of pure hepatoblastoma instead of a mixed phenotype. Lastly, we found that FTY720 acted as a sensitizer to cisplatin both *in vitro* and *in vivo*. Synergy has been observed between FTY720 and other chemotherapeutics including doxorubicin, etoposide, and cetuximab in colorectal cancer [20, 36] and cisplatin in melanoma [37]. The combination of 5-fluorouracil, SN-38, or oxaliplatin with FTY720 yielded only additive antitumor effects in colorectal cancer [19]. Alternatively, in ovarian cancer the combination of cisplatin and FTY720 behaved antagonistically due to induction of autophagy [38]. However, by inhibiting autophagy, the combination of FTY720 and cisplatin resulted in an additive effect. Since autophagy is known to play a role in hepatoblastoma [39], combining FTY720 and cisplatin with an inhibitor of autophagy like hydroxychloroquine[40] may lead to an even greater synergistic effect.

This study showed for the first time that FTY720 resulted in PP2A activation and decreased HuH6 hepatoblastoma cell viability, proliferation, and motility, increased apoptosis *in vitro*, and decreased hepatoblastoma tumor growth *in vivo*. Additionally, we have provided evidence that FTY720 behaves synergistically with cisplatin to decrease HuH6 hepatoblastoma cell proliferation and tumor growth. These findings indicate that FTY720 warrants further investigation as a novel therapeutic for the treatment of hepatoblastoma.

## Methods

### Cells and cell culture

Cell lines were maintained at 37°C and 5% CO_2_. The human hepatoblastoma cell line, HuH6, was acquired from Thomas Pietschmann (Hannover, Germany) and maintained in Dulbecco’s Modified Eagle’s Medium supplemented with 10% fetal bovine serum (HyClone, GE Healthcare Life Sciences, Logan, UT), 1 μg/mL penicillin/streptomycin (Gibco, Carlsbad, CA), and 2 mmol/L l-glutamine (Thermo Fisher Scientific, Waltham, MA). The cell line was verified within the last 12 months using short tandem repeat analysis (Heflin Center for Genomic Sciences, University of Alabama, Birmingham, Birmingham, AL), and was checked and determine to be free from *Mycoplasma*.

### Antibodies and reagents

Rabbit polyclonal anti-PP2A (ab106262) and anti-CIP2A (ab99518) were from Abcam (Cambridge, MA). Rabbit polycolonal anti-I2PP2A (sc-25564) and mouse monoclonal anti-c-myc (sc-40) were from Santa Cruz Biotechnology (Dallas, TX). Rabbit polyclonal anti-cleaved PARP (AB3565), anti-Erk1/2 (06–182), anti-phospho-Erk (Thr202/Tyr204, Thr185/Tyr187, 05-797R) were from EMD Millipore (Burlington, MA). Rabbit polyclonal anti-total PARP (#9542), anti-phospho-Akt (Ser473, #9271), and anti-Akt (#9272) were from Cell Signaling Technology (Beverly, MA). Mouse monoclonal anti-β-actin (A1978) was from Sigma Aldrich (St. Louis, MO). FTY720 and cisplatin were obtained from Cayman Chemical (Ann Arbor, MI).

### Immunoblotting

Whole-cell lysates were isolated using radioimmunoprecipitation assay (RIPA) buffer (for most immunoblots) or mTOR buffer (for phospho-Akt, Akt, phospho-Erk, and Erk immunoblots) supplemented with protease inhibitors (Sigma Aldrich), phosphatase inhibitors (Sigma Aldrich), and phenylmethanesulfonylfluoride (Sigma Aldrich). Lysates were centrifuged at 14000 rpm for 30 minutes at 4°C. Protein concentrations were determined using Pierce BCA Protein Assay reagent (Thermo Fisher Scientific) and separated by electrophoresis on ExpressPlus PAGE gradient gels. Antibodies were used according to the manufacturers’ recommended conditions. Molecular weight markers (Precision Plus Protein Kaleidoscope, Bio-Rad, Hercules, CA) were used to confirm the expected size of the proteins of interest. Immunoblots were developed with Luminata Classico or Crescendo Western HRP Substrate (EMD Millipore). Blots were stripped with stripping solution (Bio-Rad) at 65°C for 20 minutes and then re-probed with selected antibodies. Equal protein loading was confirmed using β-actin.

### Cell viability and proliferation

Cell viability was measured using the alamarBlue Cell Viability Assay (Thermo Fisher Scientific). HuH6 cells (1.5 x 10^4^ per well) were plated in 96-well plates, allowed to attach overnight, and treated with FTY720 at increasing concentrations for 24 hours. Following treatment, 10 μL of alamarBlue reagent was added to each well and the absorbance was read at 562 nm (reduced reagent) and 595 nm (oxidized reagent) using a microplate reader (BioTek Gen5, BioTek, Winooski, VT). After subtracting background absorbance of the media alone, reduction of alamarBlue reagent was calculated according to the manufacturer’s protocol. Viability was reported as fold change.

Cell proliferation was measured using the CellTiter 96 Aqueous Non-Radioactive Cell Proliferation Assay (Promega, Madison, WI). HuH6 cells (5 x 10^3^ per well) were plated in 96-well plates, allowed to attach overnight, and treated with increasing concentrations of FTY720 for 24 hours. Following treatment, 10 μL of CellTiter 96 reagent was added to each well and the absorbance was read at 490 nm using a microplate reader (BioTek Gen5) to detect the formazan product. The background absorbance of the media alone was subtracted and proliferation was reported as fold change. For the proliferation study using both FTY720 and cisplatin, the same method was utilized. Combination indices were calculated using the method of Chou-Talalay [23] and isobolograms were constructed.

### Motility

Cell migration was analyzed using a monolayer wound-healing assay. After HuH6 cells attached to the plate, a standard scratch was made in the well with a sterile 200 μL pipette tip and the cells were treated with increasing doses of FTY720. Images were obtained of the scratch wound at 0, 12, 24 and 48 hours. The area of the wound in pixels was quantified using ImageJ (https://imagej.nih.gov/ij). Data were reported as fold change scratch area and compared between groups.

### PP2A activation assay

PP2A activation was assessed following the manufacturer’s protocol for the PP2A Immunoprecipitation Phosphatase Assay Kit (EMD Millipore). Briefly, after 24 hour treatment with FTY720, HuH6 cells were lysed using NP-40 lysis buffer supplemented with protease inhibitors and phenylmethanesulfonylfluoride. Using the same amount of protein for each sample, immunoprecipitation of PP2A was performed and activity was assessed by dephosphorylation of the phosphopeptide (K-R-pT-I-R-R).

### Cell cycle analysis

HuH6 cells were treated with FTY720 (0, 6, 8, 10 μM) for 24 hours. Cells were lifted with trypsin, washed with PBS, and fixed in cold 100% ethanol for at least 30 minutes. Cells were washed with PBS and stained with a solution containing 20 μg/mL propidium iodide (Invitrogen, Carlsbad, CA) and RNAse A (0.2 mg/mL; Invitrogen) in 0.1% Triton X (Active Motif, Carlsbad, CA). Data were obtained using a FACSCalibur flow cytometer (BD Biosciences) and analyzed with FlowJo software (BD Biosciences).

### *In vivo* studies

Six-week old female athymic nude mice (Envigo, Prattville, AL) were maintained in the specific pathogen-free facility with standard 12-hour light/dark cycles and access to chow and water *ad libitum*. These specific experiments were approved by the University of Alabama at Birmingham Institutional Animal Care and Use Committee (UAB IACUC-9064) and conducted within institutional, national, international and NIH guidelines. Tumor volumes were measured with calipers and calculated with the standard formula (width^2^ x length)/2, where the length is the largest measurement. Animal weights were recorded weekly.

HuH6 human hepatoblastoma cells (2.5 x 10^6^ in 25% Matrigel; BD Biosciences) were injected subcutaneously into the right flank of the animals. When tumor size reached an average of 150 mm^3^, mice were randomized to receive vehicle (ORA-Plus, 50 μL; Perrigo, Allegan, MI) or FTY720 (10 mg/kg body weight/day in ORA-Plus, 50 μL) by oral gavage for a total of 16 days (n = 7 per group). Animals were humanely euthanized with CO_2_ and cervical dislocation 6, 12, or 24 hours after the last vehicle or drug administration, and the tumors were harvested and prepared for further study.

For the cisplatin/FTY720 combination study, HuH6 cells (2.5 x 10^6^ in 25% Matrigel, BD Biosciences) were injected subcutaneously into the right flank of 19 mice. When tumor size reached an average of 75 mm^3^, mice were randomized to receive cisplatin alone (2 mg/kg body weight/day; N = 6, Cayman Chemical), FTY720 alone (10 mg/kg body weight/day; N = 7, Selleck Chemicals), or combination treatment with cisplatin and FTY720 administered in the same doses as in the single agent groups (N = 6). Cisplatin (or sterile saline for FTY720 alone group) was administered by intraperitoneal injection on days 1–3 and 14–16. On the remaining days (days 4–13 and 17–27), FTY720 in ORA-Plus (or ORA-Plus alone for cisplatin alone group) was administered by oral gavage in a total volume of 50 μL (S2 Fig). Four hours after the last treatment dose the animals were humanely euthanized and the tumors harvested.

### Immunohistochemistry

Formalin-fixed paraffin-embedded xenograft tumor specimens were sectioned into 6 μm sections and baked at 70°C for one hour on positive slides. Slides were deparaffinized, steamed, quenched with 3% hydrogen peroxide, and blocked with blocking buffer [bovine serum albumin (BSA), powdered milk, Triton X-100, phosphate buffered saline (PBS)] for 30 minutes at 4°C. The primary antibody anti-Ki67 (rabbit polyclonal, 1:600, ab11580, Abcam) was added and incubated for 1 hour in a humidity chamber at room temperature. After washing with PBS, the secondary antibody for mouse or rabbit (Ready-to-Use biotinylated universal antibodies, Vector Laboratories, Burlingame, CA) were added for 1 hour at 22°C. The staining reaction was developed with VECTASTAIN Elite ABC reagent (PK-7100, Vector Laboratories) and Metal Enhanced DAB Substrate (Thermo Fisher Scientific). Slides were counterstained with hematoxylin. Negative controls (mouse IgG, 1 μg/mL, Invitrogen, or rabbit IgG, 1 μg/mL, EMD Millipore) were also performed. Stained slides were evaluated by a pathologist (E.M.M.) blinded to the treatment groups. The number of Ki67 cells per 500 cells in a representative section of each tumor was counted and the mean was calculated and reported [41].

### Data analysis

*In vitro* experiments were repeated with at least three biologic replicates. Data were reported as mean ± standard error of the mean (SEM). Student’s t-test or Mann-Whitney-Wilcoxon rank-sum tests were used as appropriate, with p ≤ 0.05 determined to be statistically significant.

## Supporting information

S1 Fig FTY720 increased apoptosis in hepatoblastoma cells. HuH6 cells were treated with FTY720 (0, 6, 8, 10 μM) for 24 hours. Cell cycle analysis was performed to determine the percent of cells in the sub G1 population, indicating apoptotic cells. Representative histograms are presented demonstrating an increase in the sub-G1 population following FTY720 treatment of the HuH6 cells, indicating an increase in apoptosis.
(PDF)

S2 Fig Treatment scheme for *in vivo* combination of FTY720 and cisplatin. Cisplatin (solid line) was administered by intraperitoneal injection on days 1–3 and 14–16 to mice in the cisplatin alone and combination therapy groups. For the FTY720 alone group, mice received sterile saline by intraperitoneal injection on days 1–3 and 14–16. On days 4–13 and 17–27, FTY720 in ORA-Plus (dashed line) was administered by oral gavage to the FTY720 alone and combination therapy groups. For the cisplatin alone group, mice received ORA-Plus by oral gavage on days 4–13 and 17–27.
(PDF)

S3 Fig Full length immunoblots for data set. (A) Immunoblots for PP2A, CIP2A, I2PP2A and β-actin for HuH6 cells treated with increasing doses of FTY720. (B) Immunoblots for cleaved parp, total parp and β-actin for HuH6 cells treated with increasing doses of FTY720. (C) Immunoblots for phospho-Akt, total Akt and β-actin (*top panels*); phospho-Erk, total Erk and β-actin (*middle panels*); and c-myc and β-actin (*bottom panels*) for HuH6 cells treated with increasing doses of FTY720.
(PDF)

S4 Fig Data sets. Raw data and data for graphs for Figs 1–5.
(PDF)
