# Supplementary material for: PP2A activation alone and in combination with cisplatin decreases cell growth and tumor formation in human HuH6 hepatoblastoma cells
Source: PLoS One. 2019 Apr 10;14(4):e0214469. doi: 10.1371/journal.pone.0214469 (PMC6457532; doi:10.1371/journal.pone.0214469)
Supplement: S1 Fig — HuH6 cells were treated with FTY720 (0, 6, 8, 10 μM) for 24 hours. Cell cycle analysis was performed to determine the percent of cells in the sub G1 population, indicating apoptotic cells. Representative histograms are presented demonstrating an increase in the sub-G1 population following FTY720 treatment of the HuH6 cells, indicating an increase in apoptosis. (PDF) [file pone.0214469.s001.pdf]

S1 Fig

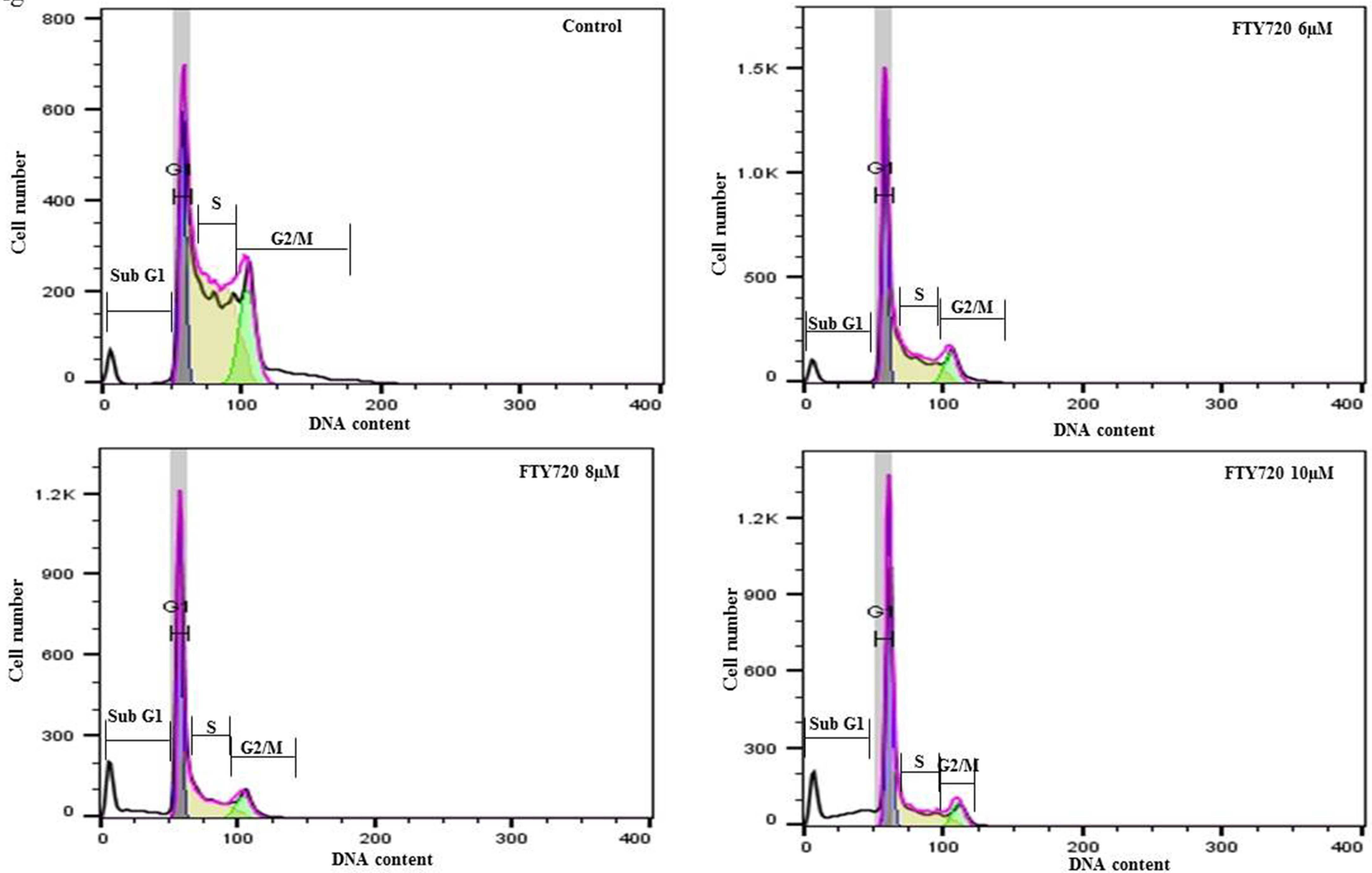
Control
FTY720 6μM
FTY720 8μM
FTY720 10μM
Cell number
DNA content
Sub G1
G1
S
G2/M
800
600
400
200
0
1.5K
1.0K
500
1.2K
900
300
100
300
400

S1 Fig FTY720 increased apoptosis in hepatoblastoma cells. HuH6 cells were treated with FTY720 (0, 6, 8, 10 μM) for 24 hours. Cell cycle analysis was performed to determine the percent of cells in the sub G1 population, indicating apoptotic cells. Representative histograms are presented demonstrating an increase in the sub-G1 population following FTY720 treatment of the HuH6 cells, indicating an increase in apoptosis.
