# Supplementary material for: PP2A activation alone and in combination with cisplatin decreases cell growth and tumor formation in human HuH6 hepatoblastoma cells
Source: PLoS One. 2019 Apr 10;14(4):e0214469. doi: 10.1371/journal.pone.0214469 (PMC6457532; doi:10.1371/journal.pone.0214469)
Supplement: S2 Fig — Cisplatin (solid line) was administered by intraperitoneal injection on days 1–3 and 14–16 to mice in the cisplatin alone and combination therapy groups. For the FTY720 alone group, mice received sterile saline by intraperitoneal injection on days 1–3 and 14–16. On days 4–13 and 17–27, FTY720 in ORA-Plus (dashed line) was administered by oral gavage to the FTY720 alone and combination therapy groups. For the cisplatin alone group, mice received ORA-Plus by oral gavage on days 4–13 and 17–27. (PDF) [file pone.0214469.s002.pdf]

S2 Fig

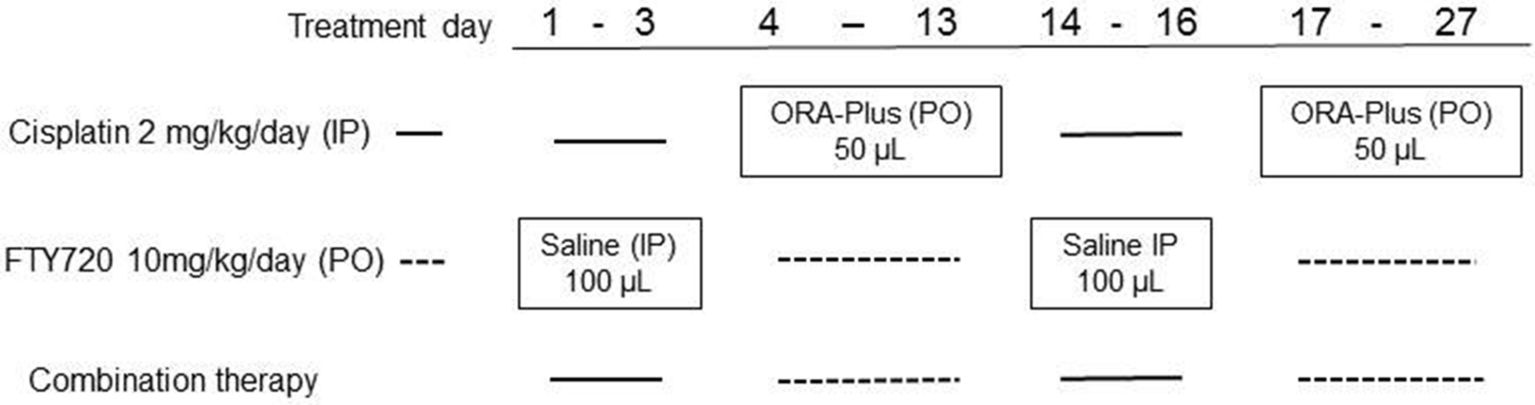
Treatment day
1 - 3
4 – 13
14 - 16
17 - 27
Cisplatin 2 mg/kg/day (IP)
ORA-Plus (PO) 50 µL
ORA-Plus (PO) 50 µL
FTY720 10mg/kg/day (PO)
Saline (IP) 100 µL
Saline IP 100 µL
Combination therapy

S2 Fig Treatment scheme for *in vivo* combination of FTY720 and cisplatin. Cisplatin (solid line) was administered by intraperitoneal injection on days 1-3 and 14-16 to mice in the cisplatin alone and combination therapy groups. For the FTY720 alone group, mice received sterile saline by intraperitoneal injection on days 1-3 and 14-16. On days 4-13 and 17-27, FTY720 in ORA-Plus® (dashed line) was administered by oral gavage to the FTY720 alone and combination therapy groups. For the cisplatin alone group, mice received ORA-Plus® by oral gavage on days 4-13 and 17-27.
