# Supplementary material for: PP2A activation alone and in combination with cisplatin decreases cell growth and tumor formation in human HuH6 hepatoblastoma cells
Source: PLoS One. 2019 Apr 10;14(4):e0214469. doi: 10.1371/journal.pone.0214469 (PMC6457532; doi:10.1371/journal.pone.0214469)
Supplement: S3 Fig — (A) Immunoblots for PP2A, CIP2A, I2PP2A and β-actin for HuH6 cells treated with increasing doses of FTY720. (B) Immunoblots for cleaved parp, total parp and β-actin for HuH6 cells treated with increasing doses of FTY720. (C) Immunoblots for phospho-Akt, total Akt and β-actin (top panels); phospho-Erk, total Erk and β-actin (middle panels); and c-myc and β-actin (bottom panels) for HuH6 cells treated with increasing doses of FTY720. (PDF) [file pone.0214469.s003.pdf]

S3 Fig (A)

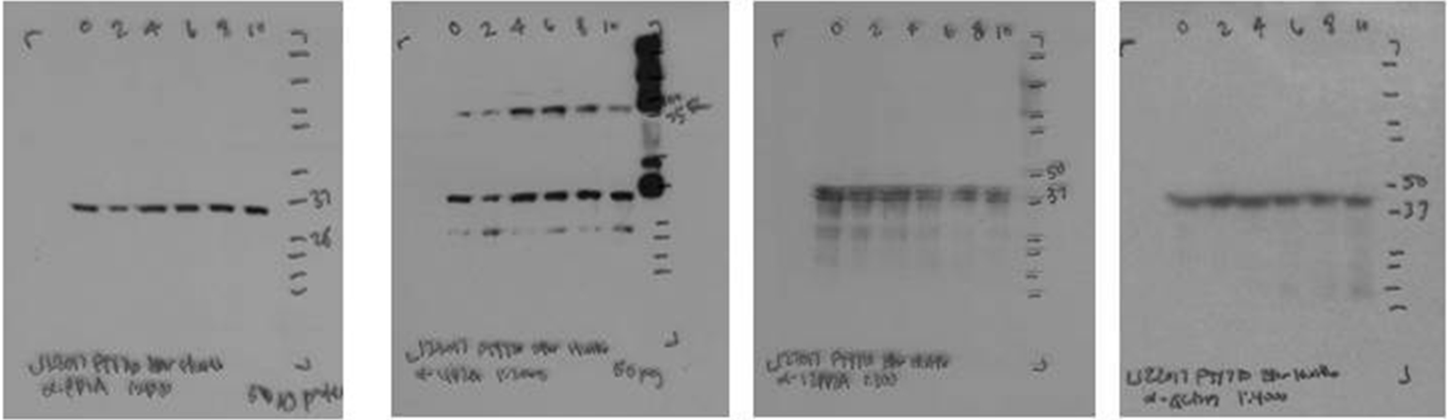

S3 Fig (B)

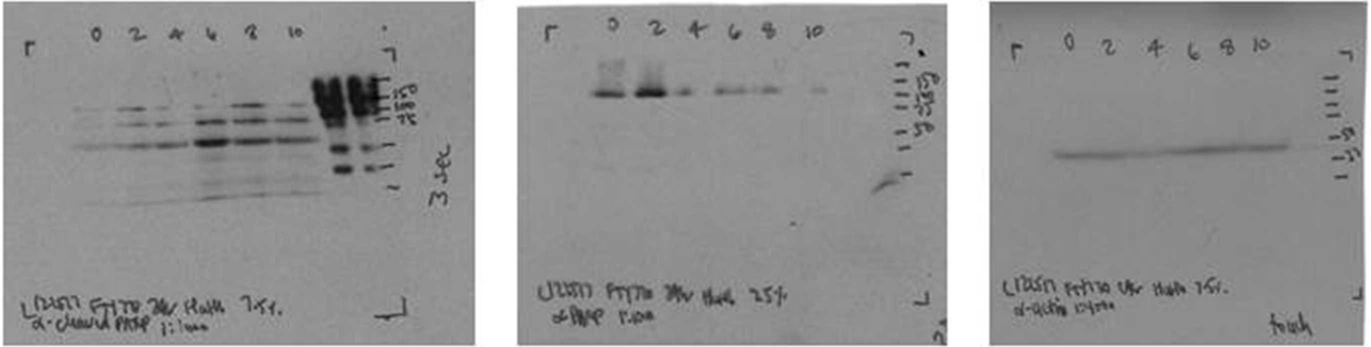

S3 Fig (C)

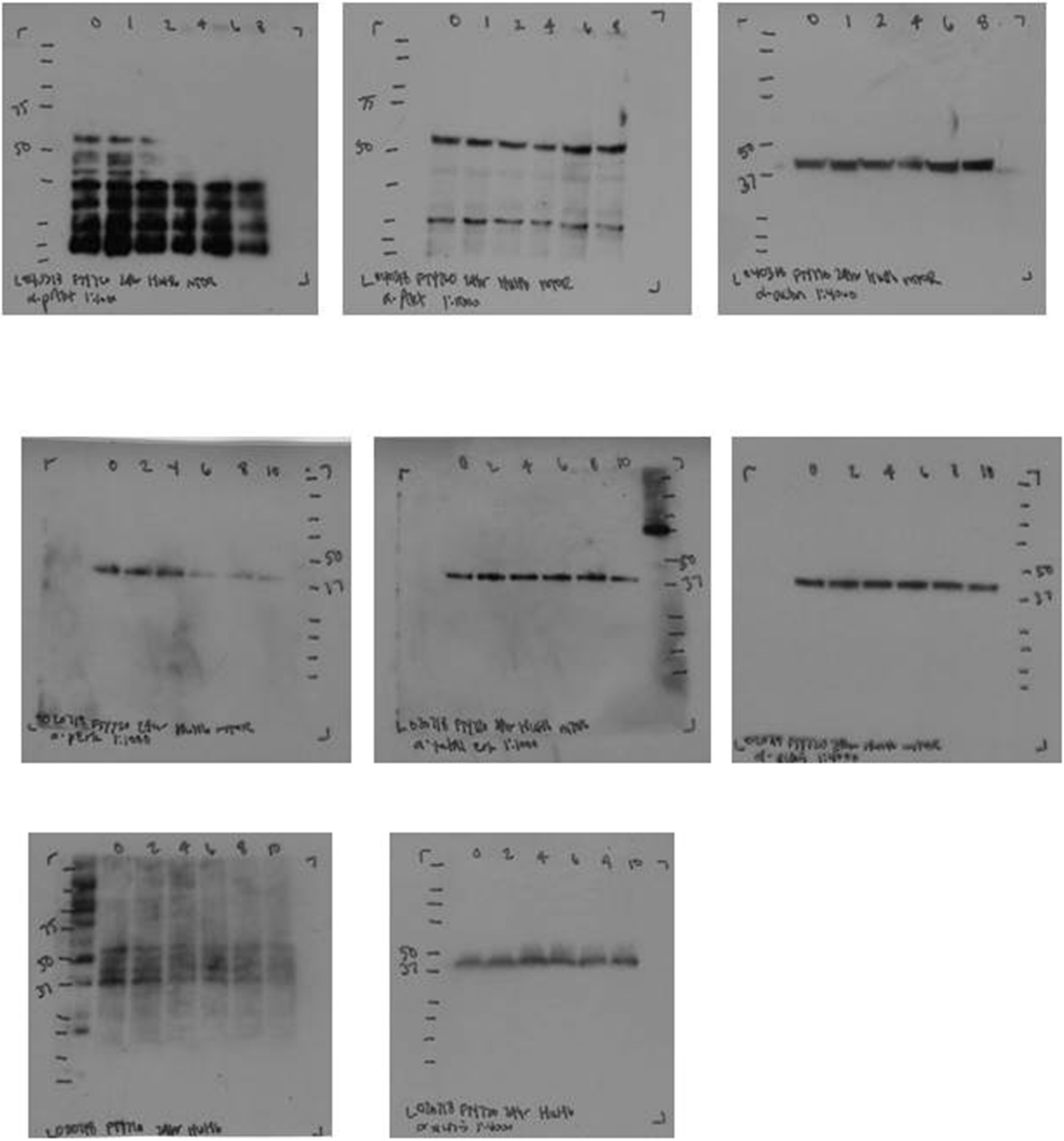

S3 Fig Full length immunoblots for data set. (A) Immunoblots for PP2A, CIP2A, I2PP2A and β-actin for HuH6 cells treated with increasing doses of FTY720. (B) Immunoblots for cleaved parp, total parp and β-actin for HuH6 cells treated with increasing doses of FTY720. (C) Immunoblots for phospho-Akt, total Akt and β-actin (*top panels*); phospho-Erk, total Erk and β-actin (*middle panels*); and c-myc and β-actin (*bottom panels*) for HuH6 cells treated with increasing doses of FTY720.
