# Supplementary material for: PP2A activation alone and in combination with cisplatin decreases cell growth and tumor formation in human HuH6 hepatoblastoma cells
Source: PLoS One. 2019 Apr 10;14(4):e0214469. doi: 10.1371/journal.pone.0214469 (PMC6457532; doi:10.1371/journal.pone.0214469)
Supplement: S4 Fig — Raw data and data for graphs for Figs 1–5. (PDF) [file pone.0214469.s004.pdf]

raw data PP2a assay

FTY720 (uM	0	2	4	6	8	10
121717	1	1.246914	1.634119	1.476992	1.493827	1.347924
121717	1	1.11254	1.476956	1.498392	1.640407	1.245177
92417	1	1.014104	1.028209	1.123413	1.271509	1.51481

Data for graph Fig 1B

	0	2	4	6	8	10
% PP2A act	100	112.4	138	136.6	146.8	136.9
SEM	0	6.7	18.2	12.2	10.7	7.8

raw data Fig 2A

uMFTY720	0	2	4	6	8	10
80917	1	1.048493	1.018832	0.992467	0.943032	0.379002
81317	1	1.02598	1.016864	0.9567	0.73526	0.220146
81917	1	1.0317	1.03122	0.979827	0.785783	0.445245

data for graph Fig2A

uMFTY720	0	2	4	6	8	10
	1	1	1	0.98	0.82	0.35
	0	0.007	0.004	0.01	0.06	0.07

raw data Fig 2B

uMFTY720	0	2	4	6	8	10
80917	1	0.860936	0.781922	0.581542	0.7268	0.088496
81317	1	0.879336	0.717195	0.616893	0.365008	0.126697
82417	1	0.862325	0.692498	0.525969	0.446	0.136026

Data for graph

uM FTY720	0	2	4	6	8	10
	1	0.87	0.73	0.57	0.51	0.12
	0	0.008	0.02	0.03	0.11	0.01

raw data Fig 2C

AZD1208 c	Time 0809	Fold Chang	AZD1208 c	Time 0824	Fold Chang	AZD1208 c	Time 1125	Fold Chang
0	0	1	0	0	1	0	0	1
0	24	0.759924	0	24	0.631008	0	24	0.670952
0	48	0.355359	0	48	0.221901	0	48	0.290059
0	72	0.08617	0	72	0.002991	0	72	0.055359
10	0	1	10	0	1	10	0	1
10	24	0.680116	10	24	0.665544	10	24	0.718363
10	48	0.631134	10	48	0.252316	10	48	0.351658
10	72	0.499398	10	72	0.027765	10	72	0.099968
20	0	1	20	0	1	20	0	1
20	24	0.732284	20	24	0.596523	20	24	0.720912
20	48	0.513099	20	48	0.256894	20	48	0.340939
20	72	0.276287	20	72	0.043745	20	72	0.119138

Data for graph Fig 2C

AZD1208 c	Time	Average	SEM
0	0	1	0
0	24	0.687295	0.038101
0	48	0.289106	0.038529
0	72	0.048173	0.024279
10	0	1	0
10	24	0.688008	0.01575
10	48	0.411703	0.113402
10	72	0.209044	0.146666
20	0	1	0
20	24	0.68324	0.043482
20	48	0.370311	0.075404
20	72	0.14639	0.068498

e

Raw data Fig 2E

Untreated	Sub G1		
1	4.04		
2	3.34		
3	3.1		
6uM FTY			
1	8.43		
2	8.77		
3	7.15		
8uM FTY			
1	18.4		
2	19.6		
3	15.9		
10uM FTY			
1	27.4		
2	30.2		
3	31.2		
data for graph Fig 5E			
uM FTY			
0	6	8	10
3.5	8.1	18	29.6
0.281977	0.493198	1.089852	1.137248

Exp 139 HuH6 FTY720

Data from experiment and for graph Fig 4A

Day of treatment				3	6	11	14	16
Days post-in	11	14	18	22	25	30	33	35
FTY720	27	35	93	67	96	149	231	360
	23	71	194	176	233	257	346	502
	17	15	81	68	76	163	432	432
	40	42	73	111	152	161	252	353
	44	63	154	120	200	258	459	760
	62	97	219	268	308	650	908	1097
	32	49	117	127	113	155	353	372
	35	53.14286	133	133.8571	168.2857	256.1429	425.8571	553.7143
	5.723802	10.07692	21.6674	26.42999	31.53888	68.06245	86.39318	105.3377

Days post-in	11	14	18	22	25	30	33	35
	45	110	282	287	588	968	1507	2176
	19	35	78	267	437	624	1231	1850
	33	58	134	202	334	588	856	807
Control	34	22	106	87	254	383	617	607
	65	89		346	488	941	1521	1813
	43	82	133	111	124	181	251	238
	68	83	168	282	669	760	994	1207
AVG	43.85714	68.42857	150.1667	226	413.4286	635	996.7143	1242.571
sem	6.663435	11.88207	29.10489	36.53439	71.94636	108.2436	176.6243	274.7185

Raw data and data for graph Fig 4B

Treatment	nor Weight (g)
	0.2
	0.5
	0.2
FTY720	0.3
	0.4
	0.6
	0.2
AVG	0.342857
sem	0.061168

Treatment	nor Weight (g)
	1.2
	0.8
	0.5
Control	0.5
	1.4
	0.1
	0.9
AVG	0.771429
sem	0.168628

Raw data and graph data for Fig 4C

Treatment	Days post-in	11	35
		22.6	23.8
		21.6	20.1
		22	21.7
FTY720		22.6	20
		20.5	20.4
		20.4	21.2
		22.7	22.5
	AVG	21.77143	21.38571
	sem	0.372069	0.528893

Treatment	Days post-in	11	35
		22.4	23.2
		19.9	18.8
		21.2	22.9
Control		21.9	21.6
		21.6	21.6
		21.6	22
		21.4	20.9
	AVG	21.42857	21.57143
	sem	0.29335	0.550634

Ki67 staining Fig 4D

	vehicle	FTY720
Raw data	20	64
	60	70
	110	30
	64	105
	97	47
		5
		5
data graph	vehicle	FTY720
	70.2	46.6
	15.8	13.8

Raw data and graph data for Fig 5B

Platin	0	0	0	0	0	0	1	0.993
	0	0	0	0	1	2.188	1.584	1.742
	0	0	1	2	1.673	2.304	4.0326	5.15222
	0	0	1	0.9912	1.447	2.0102	3.9561	4.0263
	0	0	0	1	1.182	1.626	2.477	3.387
	0	0	0	0	0	1	1.303	1.333
mean	0	0	0.333333333	0.6652	0.883666667	1.521366667	2.392116667	2.772253333
sem	0	0	0.210818511	0.3330431	0.294655807	0.360240699	0.545333265	0.681014008
combo	0	0	0	0	0	1	0.9483	1.5603
	0	0	0	0	0	0	0	1
	0	0	0	0	0	1	1.404	1.904
	0	0	1	1.255	1.1634	1.183	1.327	1.876
	0	0	0	1	0.8919	0.9122	1.068	1.784
	0	0	0	0	0	1	1.033	1.688
mean	0	0	0.166666667	0.375833333	0.34255	0.8492	0.963383333	1.635383333
sem	0	0	0.166666667	0.239966722	0.219464663	0.173661932	0.205790039	0.137144232
FTY	0	0	0	1	1.263	1.712	2.917	6.083
	0	0	1	1.511	1.977	3	3.744	4.549
	0	0	1	2.155	1.942	2.447	2.544	3.33
	0	0	0	0	0	0	1	1.037
	0	0	0	1	0.834	1.357	1.749	2.296
	0	0	0	1	1.754	1.764	4.164	5.845
	0	0	0	1	1.342	1.424	1.889	2.914
mean	0	0	0.285714286	1.095142857	1.301714286	1.672	2.572428571	3.722
sem	0	0	0.199204768	0.265192847	0.288043083	0.384801031	0.460761981	0.760383821

1.296	1.627	1.66
2.188	3.346	4.099
6.609	7.9022	10.163
8.0877	12.654	11.333
3.774	4.4968	5.368
2.324	2.636	2.717
4.04645	5.443666667	5.89
1.109928506	1.690956252	1.626097291
1.879	2.457	2.759
1.118	0.99	1.755
2.938	3.3421	2.5263
1.98	2.327	2.203
2.014	1.824	1.959
2.606	3.033	4.574
2.089166667	2.32885	2.629383333
0.257716694	0.345751427	0.416522245
6.08	6.08	7.08
5.834	7.06	6.654
4.757	7.388	6.563
1.259	1.412	0.917
3.558	4.427	4.442
9.9	10.682	14.964
3.043	3.419	3.709
4.918714286	5.781142857	6.332714286
1.127793414	1.234099006	1.787319715

Raw data and graph data Fig 5C

	Days post-in	12	42
		22	22.5
		21	21.6
		20	19.2
Cisplatin			
		25	23.1
		19.6	19.1
		20.7	20.8
	AVG	21.38333	21.05
	sem	0.79934	0.680563

	Days post-in	12	42
		19.1	21.7
		20.8	20.8
		21.9	21.9
Combinatior		21	18.5
		24.8	26.2
		19.2	18.7
	AVG	21.13333	21.3
	sem	0.856997	1.145717

	Days post-in	12	42
		21.1	
		20.8	19.5
		21.7	19.8
FTY720		21	23.1
		22.5	23.4
		20.9	22.7
		21.5	22.7
	AVG	21.35714	21.86667
	sem	0.226629	0.710243

S4 Fig Data sets. Raw data and data for graphs for Fig 1-5.
